# Comparison of Ischemic and Hemorrhagic Stroke in the Medical Ward of Dessie Referral Hospital, Northeast Ethiopia: A Retrospective Study

**DOI:** 10.1155/2021/9996958

**Published:** 2021-06-28

**Authors:** Hussen Abdu, Fentaw Tadese, Girma Seyoum

**Affiliations:** ^1^Department of Anatomy, School of Medicine, College of Health Sciences, Addis Ababa University, Addis Ababa, Ethiopia; ^2^Department of Epidemiology and Biostatistics, School of Public Health, College of Medicine and Health Sciences, Wollo University, Dessie, Ethiopia

## Abstract

**Background:**

Distinguishing the category of stroke plays a vital role in planning patient care. Simple clinical findings help distinguish the type of stroke. However, there is a need for diagnostic imaging. In Ethiopia, stroke is the most common neurological condition in patients admitted to hospitals. Yet, there are limited data on comparisons of stroke subtypes. Thus, this study was designed to determine the prevalence of stroke and to compare ischemic and hemorrhagic strokes.

**Methods:**

A retrospective cross-sectional study design was employed. Medical records containing complete information and confirmed diagnosis using imaging techniques were included. The data were entered into SPSS version 24.0 for analysis. Results with a *P* value of <0.05 were considered statistically significant.

**Results:**

From a total of 312 stroke patients, 204 (65.4%) patients were admitted due to ischemic stroke. More females, 59 (18.9%), were admitted for hemorrhagic stroke than males. In both ischemic, 175 (56.1%) and hemorrhagic, 91 (29.2%) stroke cases, most of the patients were 45 years and above. Middle cerebral artery territory was the most common site of arterial territory infarctions in ischemic stroke, 158 (50.7%). Middle cerebral artery territory also was the most common site of hematoma in hemorrhagic stroke, 91 (29.2%). Infarctions in more than one lobe of the cerebrum (16.4%) and intracerebral hemorrhage in multiple areas of the cerebrum (7.4%) were observed in ischemic as well as hemorrhagic stroke cases. Most of the ischemic, 124 (39.8%), and hemorrhagic, 39 (12.5%), stroke patients presented loss of sensation and weakness of body parts. Hypertension was observed in 124 (39.8%) ischemic and 73 (23.4%) hemorrhagic stroke patients. The mortality rate of ischemic stroke, 47 (15.3%), was two times higher than hemorrhagic stroke, 20 (6.5%). Hypertension was the most common predictor of death in both ischemic and hemorrhagic stroke cases.

**Conclusions:**

Ischemic stroke is a common type of stroke in the medical ward of the study hospital. More females were affected by hemorrhagic stroke than males. Middle cerebral artery territory was the most affected area of the brain in both ischemic and hemorrhagic strokes. Most ischemic and hemorrhagic stroke patients were admitted due to loss of sensation and weakness of body parts. Hypertension was the most common risk factor of stroke as well as a predictor of stroke-related deaths. Identification of the stroke subtypes may be important in the management of stroke. Thus, health professionals, government officials, community leaders, and the population at large could be involved in creating awareness about antecedent risk factors and clinical presentations of stroke subtypes.

## 1. Introduction

Stroke or cerebrovascular accident (CVA) is a highly heterogeneous disorder with distinct subtypes, each presenting specific clinical and epidemiological aspects [1, 2].

There are two main types of strokes. The most common type of stroke is an ischemic stroke (IS) which covers 85% of the cases produced by a blockage of blood vessels. The other less common type which covers about 15% of cases of stroke is caused by bleeding in or around the brain which is called a hemorrhagic stroke (HS) [3].

Distinguishing the category of stroke plays a vital role in planning patient care. Simple clinical findings are helpful in distinguishing the type of stroke [4, 5]. Also, the mean Glasgow Coma Scale (GCS) score in IS patients is higher than in HS patients [4].

Acute onset of headache is the most common symptom seen in HS patients compared to IS patients. In addition, computed tomography (CT) scan reports of IS patients showed hypodense lesion, hyperdense artery sign, sulcus effacement, and mass effect. However, in HS patients, a hyperdense lesion is visible [4].

However, the prognosis of victims depends on the type of stroke, the degree and duration of obstruction or hemorrhage, and the extent of brain tissue death.The location of HS is an important factor in the outcome, and this type generally has a worse prognosis than IS [6].

Even though simple clinical profiles help to distinguish the types of stroke, there is still a need for diagnostic imaging [4, 5]. Non-contrast CT scan is the most commonly used diagnostic imaging to distinguish two types of strokes, but it is not accessible in all hospitals and emergency departments, which may lead to loss of treatment golden time [4, 5, 7].

Having these issues, many studies described various clinical findings especially neurological signs and symptoms, and some of them presented formulas to distinguish stroke types based on clinical evaluations. These characteristics including focal or nonfocal symptoms, negative or positive symptoms, and sudden or gradual onset result in primary segregation of stroke types in the emergency department that leads to early diagnosis and treatment [4].

Hemorrhagic and ischemic strokes vary according to clinical presentations, outcome, and risk factors. The most common risk factors contributing to the differences in manifestation and outcome of stroke types are atherosclerosis, atrial fibrillation, and hyperlipidemia and these are by far more common in IS than in HS [6].

In Ethiopia, stroke is the most common neurological condition in patients admitted to hospitals, accounting for 24% of all neurological admissions [8–10]. Moreover, the prevalence of risk factors for stroke has been increasing in Ethiopia, due to demographic and epidemiologic shifts that affected the lifestyle of the population. There is limited research data on the clinical presentations, risk factors, and outcomes of stroke subtypes in Ethiopia [10] and in the study hospital as well. Recently updated information on the IS and HS is essential for planning, implementing, and evaluating effective and efficient preventative acute care at the health settings and for establishing home rehabilitation programs for those patients that have developed disabilities. Therefore, the objective of our investigation was to identify and compare the clinical profile, vascular and topographic distributions, associated risk factors, and outcomes of HS and IS among patients admitted to the medical ward of Dessie Referral Hospital (DRH).

## 2. Methods and Subjects

### 2.1. Study Setting and Period

The study was conducted from January 2016 to December 2019 in the medical ward of DRH. The hospital is located in Dessie city administration, in the eastern part of the Amhara national regional state, about 401 kilometers northeast of Addis Ababa, the capital city of Ethiopia. The hospital is currently serving more than 10 million people in the Amhara region.

### 2.2. Study Design

A cross-sectional retrospective study was conducted among patients admitted to the medical ward of DRH.

### 2.3. Source and Study Populations

All stroke patients as well as all systematically selected stroke patients admitted to the medical ward of the hospital during the study period were the source and study populations, respectively.

### 2.4. Eligibility Criteria

Stroke cases confirmed using CT scan or Magnetic Resonance Imaging (MRI) and admitted to the medical ward of the hospital during the study period were included in the study. Patient records with incomplete required information and absence of imaging diagnosis were excluded from the study.

### 2.5. Sample Size and Sampling Method

The sample size was calculated using Epi Info 7 with the help of single population proportion formula considering expected proportion of mortality among stroke admitted patients taken at 13% and 95% confidence level and 3% margin of error [9]. The total expected patient flow was 1200. Based on this information, the sample size was corrected to 344. From a total of 1371 admitted stroke patients, 344 medical records were selected using simple random sampling, out of which 312 fulfilled the inclusion criteria and were included in the study.

### 2.6. Data Collection Techniques and Procedures

To maintain clarity of the data, selected medical records were reviewed and the checklist was prepared after reviewing the charts. Sociodemographic characteristics, stroke patterns, clinical presentations, arteria territory and topographic distributions, associated risk factors, and outcomes of stroke were included in the checklist. Finally, the data were collected by trained medical interns from patient records using the prepared checklists.

### 2.7. Dependent Variables

Stroke subtypes, outcomes of stroke, location of infarcts, and hematomas in the brain were belonging to the dependent variables.

### 2.8. Independent Variables

The independent variables were sociodemographic characteristics of the patients such as sex, age, marital status, religion, and residence. Risk factors of stroke-like hypertension (HTN), diabetes mellitus (DM), atrial fibrillation (AF), structural heart diseases (SHD), previous history of stroke, family history of stroke, obesity, headache, and human immunodeficiency (HIV) also were included. Moreover, the behavioral characteristics of patients consisting of smoking, alcohol intake, and chat chewing as well as arterial circulations of the brain were considered.

### 2.9. Operational Definitions

(i) Alcohol intake: any amount of alcohol consumption
(ii) Stroke: defined as “rapidly developing clinical signs of focal (or global) disturbance of cerebral function lasting longer than 24 hours unless interrupted by death with no apparent cause other than that of vascular origin” confirmed with CT scan/MRI
(iii) Disability-adjusted lived years (DALYs): years which the stroke patients lived with the disabilities and impairments caused by stroke
(iv) Stroke patients' outcomes:
  (i) Complete resolution from neurological deficit: it is when an individual stroke patient is completely free of neurological deficits at the time of discharge
  (ii) Discharged with neurologic deficit: a stroke patient with improved signs and symptoms but discharged with stroke complications, especially physical impairment, cognitive impairment, and communication impairment
  (iii) Death: when loss of life occurs because of stroke and its complications
  (iv) Discharged against medical advice: it is when stroke patients refuse all the medical advice despite their health status and treatment outcomes
  (v) Glasgow Coma Scale: it helps to measure the level of consciousness [11]:
    - Good GCS (13–15): mild brain injury (alert)
    - Moderate GCS (9–12): moderate brain injury (drowsy)
    - Poor GCS (≤8): severe brain injury (unconscious)

## 3. Data Analysis

The obtained data were checked, cleaned, and entered into Statistical Package for Social Science (SPSS) version 24.0 software for analysis. Study participants were described using frequency, proportion, and summary measures. Binary and multivariable logistic regressions were done to identify factors associated with the dependent variables. The results were presented in the form of texts, tables, and figures. Results with a *P*value of <0.05 were considered statistically significant.

## 4. Results

### 4.1. Sociodemographic Characteristics of Patients

In the current study, all the study cases were confirmed using either CT scan, 239 (76.6%), MRI, 50 (16.0%), or both CT scan and MRI, 23 (7.4%), investigations.

**Figure 1:**
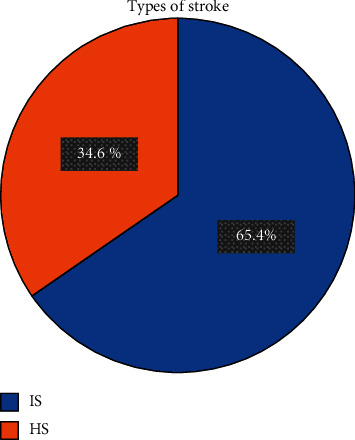
Proportion of stroke subtypes. HS: hemorrhagic stroke; IS: ischemic stroke.

Out of 312 study participants, 204 (65.4%) patients were admitted due to IS, whereas the remaining 108(34.6%) patients were admitted because of HS (Figure 1). Of the patients with IS, half of the 102 (32.7%) patients were males. However, more females 59 (18.9%) than males were admitted for HS. In both IS, 175 (56.1%), and HS, 91(29.2%) cases, most of the patients were 45 years and above. Most of the cases of both IS, 119 (38.2%), and HS, 65 (20.8%), had no formal education. In addition, nearly two-thirds of the patients, 135 (43.3%) in IS and 70(22.4%) in HS, were from rural areas. There were no statistical differences in both IS and HS in relation to the sociodemographic characteristics of the patients (Table 1).

### 4.2. Arterial Territory Distribution of Ischemic and Hemorrhagic Stroke

**Figure 2:**
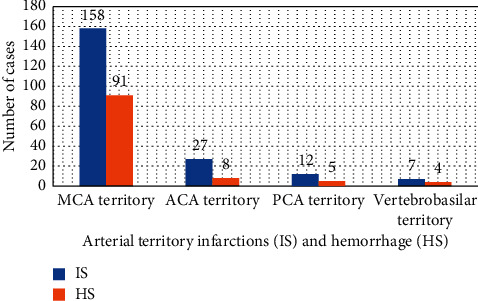
Arterial territory infarctions (IS) and hemorrhage (HS) with stroke subtypes. ACA: anterior cerebral artery, HS: hemorrhagic stroke, IS: ischemic stroke, MCA: middle cerebral artery, and PCA: posterior cerebral artery.

Middle cerebral artery (MCA) territory was the commonest site of arterial territory infarctions in IS which was seen in 158 (50.7%) patients followed by anterior cerebral artery (ACA), 27 (8.7%), and posterior cerebral artery (PCA), 12 (3.8%), territories. The vertebrobasilar arterial territories were the least affected area of the brain that was observed in 7 (2.2%) patients. Likewise, middle cerebral artery territory was also the commonest site of hematoma in HS observed in 91(29.2%) patients (Figure 2).

### 4.3. Topographic Distribution of Ischemic and Hemorrhagic Stroke

**Figure 3:**
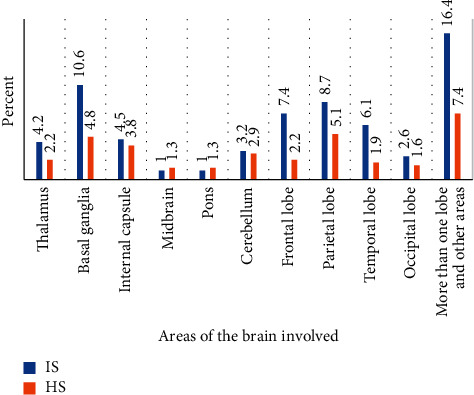
Prevalence and topographic distribution of ischemic and hemorrhagic stroke. HS: hemorrhagic stroke; IS: ischemic stroke.

In this study, infarctions in more than one lobe of the cerebrum and occurring together with infarctions in other areas (like thalamus, hypothalamus, and basal ganglia) were observed in 16.4% of patients with IS. The prevalence of infarctions in the basal ganglia and parietal lobe was 10.6% and 8.7%, respectively. Intracerebral hemorrhage in multiple areas of the brain was observed in 7.4% of the HS cases. Midbrain and pons were the least affected areas of the brain in both IS (1%) and HS (1.3%) (Figure 3).

### 4.4. Clinical Presentations

At the time of arrival to the hospital, 124(39.8%) IS and 39 (12.5%) HS patients presented loss of sensation and weakness of body parts. Ninety-six (30.8%) IS and 44 (14.1%) HS patients presented hemiplegia/hemiparesis. More HS patients experienced headache, 7 (2.2%), and vomiting, 13 (4.2%), than IS patients. The mean GCS of HS patients at the time of admission was 8.3 ± 4.6 (mean ± SD), ranging from 3 to 15, while in IS patients it was 11.7 ± 3.4, ranging from 5 to 15. The mean systolic blood pressure (SBP) and diastolic blood pressure (DBP) of IS patients (SBP = 158.7 ± 26.4 mmHg; DBP = 96.4 ± 21.3 mmHg) were higher than those in HS patients (SBP = 148 ± 22.8 mmHg; DBP = 93.2 ± 16.4 mmHg) (Table 2).

### 4.5. Antecedent Risk Factors of Stroke

In the present study, the most common risk factor identified in both IS and HS patients was HTN that was seen in 124 (39.8%) and 73 (23.4%) IS and HS patients, respectively. Atrial fibrillation, 27 (8.7%), SHD, 21 (6.7%), family history of stroke, 9 (2.9%), and HIV, 10 (3.2%), were observed more in HS patients than in IS patients (Table 3).

To get more candidate variables on binary logistic regression, a 0.2 *P*value was used as a cutoff point. Hence, DM, AF, SHD, having a family history of stroke, HIV, and headache/migraine were nominated to be included in the multivariable logistic regression. On multivariable logistic regression using 95% CI, AF (AOR: 0.39), SHD (AOR: 0.44), and family history of stroke (AOR: 0.32) were statistically significant predictor risk factors for HS at a *P*value of <0.05. Patients with AF, SHD, and family history of stroke were 0.39, 0.44, and 0.32 times less likely to develop IS than HS, respectively (Table 4).

### 4.6. Outcomes Status of Ischemic and Hemorrhagic Stroke Patients

The outcome status of four IS and one HS cases were not stated in their respective medical records. Consequently, the outcomes of 307 (200 (65.1%) IS and 107 (34.9%) HS) patients were analyzed. Seventy-one (23.1%) IS and 39 (12.7%) HS patients were discharged from the hospital with complete resolution of neurological deficits.

About 14.6% of IS and 7.5% of HS cases were against the consultation of the hospital and left without permission. The mortality rate of IS in this study was two times higher than that of HS, where 47 (15.3%) IS and 20 (6.5%) HS patients died. But, nearly the same proportion of IS 23 (7.5%) and HS 20 (6.5%) patients were discharged with neurologic deficit (Table 5).

Out of 47 (15.3%) deaths in IS, 30 (10%) of them were hypertensive and 12 (4%) of them had AF, whereas 14 (5%) and 8 (2%) deaths in HS cases were hypertensive and had SHD, respectively (Table 6).

## 5. Discussion

Clinical profile, risk factors, and prognosis of stroke patients depend on the type of stroke, the degree, location, and duration of obstruction or hemorrhage, and the extent of brain tissue death [6]. This study compared hemorrhagic and ischemic strokes in relation to clinical presentations, risk factors, and outcomes among patients admitted to the medical ward of Dessie Referral Hospital.

In this study, IS was the dominant type of stroke compared to HS. Many other studies also reported similar results where the prevalence and incidence of IS were greater than those of HS [9, 12].

Results of this hospital-based study showed that there was no sex difference in the prevalence of IS. The same proportion of males and females were admitted due to IS. This finding was inconsistent with reports of other studies where the male gender was a significantly stronger predictor of IS [13]. In our investigation, more females than males were admitted because of HS. This observation was in agreement with the reports of other studies where the female gender was found to be a stronger predictor of HS [13]. A possible explanation for more women with HS might be due to the positive effect of estrogen in the cerebral circulation that can change the physiologic activities of the body and disorders caused by alterations of the normal body functions. On the other hand, more males had IS than HS. This observed sex difference may be explained by higher prevalence of cardiovascular conditions such as higher blood pressure in men than women of similar age [14] as well as increased risk factors of stroke such as chat chewing, smoking, and alcohol intake in males.

Subjects with IS had a higher mean age (63.4 ± 9.6 years) than subjects with HS (53 ± 9.6 years). Yet, there was no statistically significant age difference in both IS and HS. This finding was consistent with those reported by other previous studies conducted in the different regions of the world and other regions of Ethiopia [4, 9, 12, 15–17].

Accurate evidence of the arterial territory infarcted after the occlusion of a specific cerebral artery or hematoma after the rapture of arteries provides pertinent information about stroke mechanism and helps the planning of investigations and succeeding therapy. Precise knowledge of arterial territory allows the distinction between infarcts/hematomas located within an arterial territory and infarcts/hematomas in the border zone between arterial territories [18–20]. In this study, MCA territory was the most common arterial territory involved in both IS and HS. The frequency of ACA territory infarctions was 27 in IS and 8 in HS cases. This observation was in agreement with the results of previous studies which also reported 27 IS cases in the Lausanne Stroke Registry [21], however, lower than the number in Barcelona, Spain, which reported 51 cases [22].

Locations of infarctions and hematomas in and on the brain depend on the arterial territory circulations. In this study, infarctions in multiple areas of the brain occurred in 16.4% of IS cases. Basal ganglia, parietal, and frontal lobes of the cerebrum were the most common sites of infarctions. Furthermore, intracerebral hematomas in multiple areas of the brain were observed in 7.4% of HS. In another study, basal ganglia (38%) were reported to be the most common site of hematomas followed by thalamus (29.6%) and lobar hemorrhage (18.3%) [8].

The most commonly found symptom in subjects with IS was loss of sensation and weakness of body parts. This finding is in agreement with other previous studies, in which arm weakness and leg weakness were found to be the most commonly complained symptoms in patients with IS [12, 23].

Hemorrhagic strokes have a wide range of clinical appearances, though acute onset of headache, vomiting, and severe increases in blood pressure are the most prevalent signs and symptoms that lead to localized neurological signs, developing in a few minutes [4, 24]. Likewise, the results of our study showed headache and vomiting as the common clinical symptoms in HS patients compared to IS patients.

The results of the present study showed a lower mean GCS in HS patients than in IS patients. This is in line with other previous studies which reported a mean GCS of 12.67 in IS patients and 8.97 in HS patients. But, the minimum scores in our study were 3 and 5 in IS and HS patients, respectively, which were slightly lower than other studies [4].

The mean systolic and diastolic blood pressures in both IS and HS were measured high and this can make hypertension a common frequently seen risk factor for both IS and HS which agreed with various population and hospital-based studies that reported HTN as the most common risk factor predisposing patients for all subtypes of stroke [9, 10, 13, 15, 25–29].

Hemorrhagic and ischemic stroke differ according to outcome and risk factors [6]. In this study, patients with HS were more likely to have AF, SHD, family history of stroke, and HIV than IS patients.

In the current study, a high percentage of patients left the hospital against medical advice. This may be related to the high percentage of patients not having formal education (38.2% in IS and 20.8 in HS patients). From other studies, in age-adjusted analyses, IS survivors without formal education or with one to seven years of education had an approximately twofold to fourfold risk of functional dependence compared to those with ≥8 years of education [30]. Thus, as secondary stroke prevention is of great importance, it is likely that these patients would be lost to follow up, increasing the risk of stroke recurrence.

Furthermore, the mortality rate of IS was two times more than that of HS. This result was inconsistent with other pieces of literature in which patients with HS have a higher mortality rate [31]. A higher number of deaths were recorded in both IS and HS patients who had HTN, AF, and SHD. There is a continual argument concerning the effect of blood pressure on the outcome of stroke. Some studies report a worse prognosis in patients with markedly elevated blood pressure [32, 33].

## 6. Conclusion and Recommendations

Ischemic stroke was the common type of stroke in our study hospital. The same proportions of males and females were admitted due to IS; however, more females were admitted for HS. Hypertension was found to be the most common risk factor in both IS and HS cases. A high rate of mortality was seen in IS cases and most of the cases were hypertensive. Identification of the stroke subtypes may be important in the management of stroke. Thus, health professionals, government officials, community leaders, and the population at large could be involved in creating awareness about antecedent risk factors and clinical presentations of stroke subtypes. In addition, establishing well-equipped emergency setup is important for patient prognosis and simplify the outcome of the patients. 

## 7. Limitations of the Study

The results of hospital-based studies could not be inferred to the general population and avoiding bias in retrospective type of study is challenging.

## Figures and Tables

**Table 1:** Sociodemographic distribution of hemorrhagic and ischemic stroke.

Variables	Categories	IS		HS		*P*value
		204	65.4%	108	34.6%	
*Sex*	Male	102	32.7	49	15.7	0.49
	Female	102	32.7	59	18.9	

*Age*	<45	29	9.3	17	5.4	0.72
	≥45	175	56.1	91	29.2	

*Marital status*	Single	3	1.0	2	0.6	0.88
	Married	153	49.1	81	26.0	
	Widowed	20	6.4	8	2.6	
	Divorced	11	3.5	5	1.6	
	Not specified	17	5.5	12	3.8	

*Religion*	Muslim	109	34.9	63	20.2	0.29
	Orthodox	75	24.0	40	12.8	
	Protestant	17	5.5	3	1.0	
	Catholic	3	1.0	2	0.6	

*Educational status*	No formal education	119	38.2	65	20.8	0.67
	Primary school	24	7.7	12	3.8	
	Secondary school	14	4.5	10	3.2	
	College and above	13	4.2	3	1.0	
	Not specified	34	10.9	18	5.8	

*Residence*	Rural	135	43.3	70	22.4	0.81
	Urban	69	22.1	38	12.2	

HS: hemorrhagic stroke; IS: ischemic stroke.

**Table 2:** Clinical presentations of ischemic and hemorrhagic stroke.

Clinical presentations	IS = 204 (65.4%)		HS = 108 (34.6)	
	No. of cases	%	No. of cases	%
Loss of consciousness	49	15.7	28	9.0
Weakness and sensory loss	124	39.8	39	12.5
Aphasia	36	11.5	27	8.7
Urinary incontinence	45	14.4	22	7.0
Hemiplegia/hemiparesis	96	30.8	44	14.1
Grasp reflex	3	1.0	2	0.6
Facial palsy	23	7.4	5	1.6
Dysarthria	3	1.0	4	1.3
Quadriplegia	2	0.6	2	0.6
Headache	3	1.0	7	2.2
Hemianopsia	4	1.3	3	1.0
Diplopia	6	1.9	3	1.0
Visual perceptual deficits	7	2.2	6	1.9
Ataxia and incoordination	12	3.8	7	2.2
Vomiting	10	3.2	13	4.2
Convulsions	3	1.0	3	1.0
SBP (mean ± SD)	158.7 ± 26.4 mmHg		148 ± 22.8 mmHg	
DBP (mean ± SD)	96.4 ± 21.3 mmHg		93.2 ± 16.4 mmHg	
GCS (mean ± SD)	11.7 ± 3.4		8.3 ± 4.6	

DBP: diastolic blood pressure; GCS: Glasgow Coma Scale; HS: hemorrhagic stroke; IS: ischemic stroke; mmHg: millimeter of mercury; SBP: systolic blood pressure; SD: standard deviation.

**Table 3:** Prevalence of antecedent risk factors of ischemic and hemorrhagic stroke.

Risk factors	Categories	IS = 204 (65.4%)		HS = 108 (34.6%)		*P*value
		No. of cases	%	No. of cases	%	
*HTN*	Yes	124	39.8	73	23.4	0.24
	No	80	25.6	35	11.2	

*DM*	Yes	11	3.5	13	4.2	0.04^*∗*^
	No	193	61.9	95	30.4	

*Previous history of stroke*	Yes	12	3.8	5	1.6	0.64
	No	192	61.6	103	33.0	

*AF*	Yes	20	6.4	27	8.65	0.01^*∗*^
	No	184	59.0	81	26.0	

*Smoking*	Yes	22	7.1	16	5.1	0.3
	No	182	58.3	92	29.5	

*Alcohol intake*	Yes	24	7.7	8	2.6	0.23
	No	180	57.7	100	32.0	

*SHD*	Yes	18	5.8	21	6.7	0.01^*∗*^
	No	186	59.6	87	27.9	

*HTN/DM*	Yes	17	5.5	13	4.2	0.29
	No	187	60.0	95	30.4	

*Family history of stroke*	Yes	6	1.9	9	2.9	0.03^*∗*^
	No	198	63.5	99	31.7	

*HIV*	Yes	7	2.2	10	3.2	0.03^*∗*^
	No	197	63.2	98	31.4	

*Chat chewing*	Yes	33	10.6	13	4.2	0.33
	No	171	54.8	95	30.4	

*Obesity*	Yes	10	3.2	6	1.9	0.8
	No	194	62.2	102	32.7	

*Headache/migraine*	Yes	10	3.2	11	3.5	0.08^*∗*^
	No	194	62.2	97	31.1	

AF: atrial fibrillation, DM: diabetes mellitus, HIV: human immunodeficiency virus, HS: hemorrhagic stroke, HTN: hypertension, IS: ischemic stroke, and SHD: structural heart diseases; ^*∗*^statistically significant risk factor for HS at *P*value <0.05.

**Table 4:** Predictors of hemorrhagic stroke.

Risk factors	Categories	IS = 204 (65.4%)		HS = 108 (34.6%)		AOR (95% CI)	*P*-value
		No. of cases	%	No. of cases	%		
*DM*	Yes	11	3.5	13	4.2	0.52 (0.21–1.26)	0.15
	No	193	61.9	95	30.4	1	

*AF*	Yes	20	6.4	27	8.7	0.39 (0.19–0.81)	0.01^*∗*^
	No	184	59	81	26	1	

*SHD*	Yes	18	5.8	21	6.7	0.44 (0.22–0.90)	0.02^*∗*^
	No	186	59.6	87	27.9	1	

*Family history of stroke*	Yes	6	1.9	9	2.9	0.32 (0.11–0.97)	0.04^*∗*^
	No	198	63.5	99	31.7	1	

*HIV*	Yes	7	2.2	10	3.2	0.44 (0.15–1.24)	0.12
	No	197	63.1	98	31.4	1	

*Headache/migraine*	Yes	10	3.2	11	3.5	0.62 (0.23–1.62)	0.33
	No	194	62.2	97	31.1	1	

AF: atrial fibrillation, AOR: adjusted odds ratio, CI: confidence interval, COR: crude odds ratio, DM: diabetes mellitus, HIV: human immunodeficiency virus, HS: hemorrhagic stroke, IS: ischemic stroke, and SHD: structural heart diseases; ^*∗*^statistically significant risk factor for HS at *P*value < 0.05.

**Table 5:** Outcome statuses of patients with stroke subtype.

Outcome statuses	IS = 200 (65.1%)		HS = 107 (34.9)	
	No. of cases	%	No. of cases	%
Discharged with complete resolution of neurological deficits	71	23.1	39	12.7
Discharged with neurological deficits	23	7.5	20	6.5
Discharged against medical advice	45	14.6	23	7.5
Referred	14	4.6	5	1.6
Died	47	15.3	20	6.5

HS: hemorrhagic stroke; IS: ischemic stroke.

**Table 6:** Prevalence of risk factors for hemorrhagic and ischemic stroke-related deaths.

Risk factors	IS		HS	
	No. of deaths	%	No. of deaths	%
HTN	30	10	14	5
DM	2	1	5	2
HTN/DM	4	1	2	1
Previous stroke	7	2	2	1
AF	12	4	7	2
Smoking	5	2	5	2
Alcohol intake	5	2	3	1
SHD	7	2	8	3
Family history of stroke	2	1	3	1
HIV	4	1	2	1
Chat	7	2	3	1
Obesity	3	1	3	1
Headache	3	1	2	1

AF: atrial fibrillation, DM: diabetes mellitus, HIV: human immunodeficiency virus, HS: hemorrhagic stroke, HTN: hypertension, IS: ischemic stroke, and SHD: structural heart diseases.

## Data Availability

All the necessary data used to support the results of this study are included in the manuscript.

## Abbreviations

ACA: Anterior cerebral artery
AF: Atrial fibrillation
AOR: Adjusted odds ratio
CI: Confidence interval
COR: Crud odds ratio
CT: Computed tomography
CVA: Cerebrovascular accident
DALYs: Disability-adjusted lived years
DAMA: Discharged against medical advice
DBP: Diastolic blood pressure
DRH: Dessie Referral Hospital
DWND: Discharged with neurologic deficit
GCS: Glasgow Coma Scale
HIV: Human immunodeficiency virus
HS: Hemorrhagic stroke
HTN: Hypertension
IS: Ischemic stroke
MCA: Middle cerebral artery
mmHg: Millimeter of mercury
MRI: Magnetic resonance imaging
PCA: Posterior cerebral artery
SBP: Systolic blood pressure
SD: Standard deviation
SHD: Structural heart diseases
SPSS: Statistical Package for the Social Sciences.
